# The Role of Cancer Stem Cells in the Organ Tropism of Breast Cancer Metastasis: A Mechanistic Balance between the “Seed” and the “Soil”?

**DOI:** 10.1155/2012/209748

**Published:** 2011-11-03

**Authors:** Jenny E. Chu, Alison L. Allan

**Affiliations:** ^1^Department of Anatomy & Cell Biology, Schulich School of Medicine and Dentistry, University of Western Ontario, London, ON, Canada N6A 3K7; ^2^London Regional Cancer Program, London Health Sciences Centre, London, ON, Canada N6A 4L6; ^3^Department of Oncology, Schulich School of Medicine and Dentistry, University of Western Ontario, London, ON, Canada N6A 4L6; ^4^Lawson Health Research Institute, Cancer Research Laboratories, London, ON, Canada N6A 4V2

## Abstract

Breast cancer is a prevalent disease worldwide, and the majority of deaths occur due to metastatic disease. Clinical studies have identified a specific pattern for the metastatic spread of breast cancer, termed organ tropism; where preferential secondary sites include lymph node, bone, brain, lung, and liver. A rare subpopulation of tumor cells, the cancer stem cells (CSCs), has been hypothesized to be responsible for metastatic disease and therapy resistance. Current treatments are highly ineffective against metastatic breast cancer, likely due to the innate therapy resistance of CSCs and the complex interactions that occur between cancer cells and their metastatic microenvironments. A better understanding of these interactions is essential for the development of novel therapeutic targets for metastatic disease. This paper summarizes the characteristics of breast CSCs and their potential metastatic microenvironments. Furthermore, it raises the question of the existence of a CSC niche and highlights areas for future investigation.

## 1. Introduction

Due to the expanding and aging global population, it is no surprise that cancer incidence and mortality are increasing despite ongoing research in the areas of cancer treatment and prevention. In North American women, breast cancer represents the most commonly diagnosed and the second highest cause of cancer-related deaths [1, 2]. Although the collection of exact global cancer statistics is difficult due to differences in healthcare infrastructure and data collection methods, the GLOBOCAN study ranks breast cancer as the most frequently diagnosed and the most prevalent cause of cancer-related death among women globally [3]. In the past, breast cancer has been a higher burden in developed countries, likely due to more risk factors associated with lifestyle such as postponement of pregnancy until after 30, less breast-feeding, smaller families, and a less active workplace [4]. It is predicted that as developing countries improve their economic conditions and adopt a more “westernized” lifestyle, incidence rates will increase [5]. The challenge then presents itself: what is the best way to target this lethal disease in developed countries while also counteracting the predicted increase in mortality in developing countries? The answer lies in the understanding of metastatic disease, the most lethal aspect of breast cancer.

## 2. Metastasis

Even though advances have been made in prevention, detection, and treatment, the mortality rate associated with breast cancer has remained high [3]. Primary breast tumors originate within the lobule or duct of the breast, and therapies are highly efficient if the neoplasm is detected while localized within the original structure (*in situ*) or even still localized within the breast itself [6]. Therapeutic efficacy is greatly reduced once the cancer acquires invasive and metastatic properties. Therefore, metastatic disease represents the aspect of breast cancer responsible for the majority of breast-cancer-related mortalities.

Following successful angiogenesis at the primary tumor site, the stepwise process of metastasis has been clearly defined. During the initial stage, cells escape from the primary tumor into the blood and/or lymphatic system via a process called intravasation. Once in the circulation, these cells must survive until they reach a secondary site where they arrest and enter the tissue (extravasation). Tumor cells able to initiate and maintain colony growth in these secondary sites form micrometastases which, following angiogenesis, grow into clinically detectable macrometastases [7–9].

## 3. Metastatic Theories

Clinical observations highlight that different cancers exhibit characteristic sites for secondary metastases that are dependent on the origin of the primary tumor, a phenomenon termed organ tropism [10, 11]. For example, breast cancer preferentially spreads to the lymph nodes, lung, liver, bone, and brain, while other primary cancers have different preferential sites of metastasis (i.e., prostate cancer and colorectal cancer spread to bone and liver, respectively) [11]. While there are many theories concerning the mechanisms of metastasis (eloquently summarized by Hunter et al. [12]), only a few sufficiently account for the organ tropism phenomenon. Two of the main theories that have been proposed to explain this organ tropism of cancer metastasis include the “seed and soil” theory, first documented by Stephen Paget in 1889, and Ewing's mechanical arrest theory [13, 14]. Paget postulated that organ-specific patterns could be accounted for by the needs of the cancer cell (the seed) for a specific environment (the soil) in order to initiate and maintain growth [13]. Ewing's theory, proposed thirty years later, postulates that organ tropism can be accounted for by circulatory patterns within the body and that cells are mechanically arrested in the first capillary bed they encounter [14]. It is likely that these two theories are not mutually exclusive, but rather that they work in concert to produce successful metastases: cells arrest due to mechanical obstruction and/or specific chemical signals and then require a suitable microenvironment for initiation and maintenance of secondary tumor growth.

An autopsy study by Dr. Leonard Weiss [10] addressed the differences between the “seed and soil” and mechanical arrest theories by not only investigating the incidence of metastatic lesions at secondary sites, but by also taking into account the innate blood flow to each of the sites. This study used these two parameters to generate a “metastatic efficiency index” (MEI) that was used to rank pairs of primary and secondary sites as either accounted for by blood flow alone, or as “friendly” (more incidence than suggested by blood flow patterns alone) versus “hostile” (less incidence than dictated by blood flow) interactions. Interestingly, 66% of the pairs could be attributed to blood flow due to the sheer number of cancer cells delivered to the sites in arterial blood (i.e., mechanical arrest), while 20% of pairs were ranked as “friendly” and 14% of pairs were deemed to be due to “hostile” interactions. Of note, prostate and breast cancer were seen to exhibit a “friendly” interaction with bone; while ovarian, prostate, stomach, and urinary bladder cancers were seen to have a “hostile” interaction with the brain [10] (Table 1). This study suggests that some site-specific metastases can be attributed to blood flow patterns, but that there is also a distinct seed and soil effect for others. The question of whether the properties of the secondary organ or the properties of the cancer cell are more important in mediating the organ tropism of breast cancer remains to be answered. 

## 4. Metastatic Inefficiency

Although often lethal when successful, the multistep nature of the metastatic process lends itself to a high degree of inefficiency. In an experimental mouse model, Luzzi et al. used *in vivo* videomicroscopy to demonstrate that only 0.02% of melanoma cells injected intraportally to target the liver could successfully complete the entire metastatic process [15]. Interestingly, the authors noted that not all metastatic stages are equally inefficient, but rather that the main inefficiencies occur during the initiation and maintenance of metastatic lesions in the secondary organ. Many tumor cells are capable of extravasating into the secondary site, but may become dormant due to lack of external growth signals [16], and/or may fail to colonize the site due to a lack of ability to recruit sufficient blood supply to support the formation of a clinically relevant lesion.

This inefficiency appears to be mirrored in humans as, in a limited study of palliative ovarian cancer patients, ascites fluid full of tumor cells that was shunted directly into the venous circulation via peritoneovenous shunts did not always cause secondary lesions. Some but not all of these cases resulted in pulmonary metastases, although these lesions were clinically irrelevant as patient mortality resulted first from primary tumor progression. Other cases did not develop detectable metastatic lesions within the timeframe of the study (up to 27 months) before they too succumbed to their original tumor [17]. Both murine and human studies suggest that only a rare subpopulation of primary tumor cells can successfully complete the metastatic process, and likely the outcome also depends on the secondary organ microenvironment. Our group and others hypothesize this rare subpopulation of tumor cells to be cancer stem cells (CSCs) [18–21].

## 5. Cancer Stem Cells

The composition of primary breast tumors has been shown to be heterogeneous with respect to both molecular subtype (luminal A, luminal B, basal-like, HER2-overexpressing, normal breast-like, and claudin-low) [22, 23] and cellular function, even within the same tumor [24, 25]. This heterogeneity can be accounted for by the CSC hypothesis, also known as the hierarchy theory, which posits that there is a small, phenotypically identifiable subpopulation of cancer cells with stem cell-like characteristics [26]. These CSCs sit at the top of this functional hierarchy and are postulated to be capable of tumor propagation and maintenance due to their ability to self-renew and to differentiate into the cells comprising the bulk of the tumor. Conversely, the terminally differentiated non-CSCs are not capable of producing large amounts of progeny or of tumor propagation [25, 27, 28]. 

The first identification of CSCs in solid tumors came from the seminal work of Dr. Michael Clarke's group [29] following the lead of Dr. John Dick and colleagues in the leukemia field [30]. Working with cells isolated from the pleural effusions and primary tumors of breast cancer patients, Al-Hajj et al. [29] isolated distinct subpopulations of tumor cells using fluorescence-activated cell sorting. The epithelial-specific antigen positive (ESA^+^) CD44^+^ CD24^−/low^ lineage negative (Lin^−^) subpopulation was capable of forming tumors when as few as 100 cells were injected into the mammary fat pad of nonobese diabetic/severe combined immune deficiency (NOD/SCID) mice, whereas tens of thousands of cells from other subpopulations were nontumorigenic. Ginestier et al. [31] further purified this breast CSC subpopulation by adding in the criteria of high aldehyde dehydrogenase activity (ALDH^hi^). ALDH^hi^ CD44^+^ CD24^−^ breast tumor cells were capable of tumor initiation when as few as 20 cells were injected into NOD/SCID mice. These tumors exhibited the same phenotypic heterogeneity as the initial tumors, exhibiting both tumorigenic and nontumorigenic subpopulations. Furthermore, this tumor formation and heterogenic recapitulation could be replicated upon serial passaging in naïve NOD/SCID mice of the ALDH^hi^ CD44^+^ CD24^−^ cells isolated from tumors derived from the initial CSC injection, demonstrating the CSCs' differentiation and self-renewal potential [31]. 

Breast CSCs demonstrate an increased metastatic propensity *in vitro *[18, 32, 33], *in vivo *[18, 21, 34], and in clinical observation [20, 35]. Although their metastatic role is not fully understood, many theories have attempted to explain the contribution of CSCs to breast cancer metastasis. The most common site of breast cancer metastasis is to the bone, but metastatic lesions are also found in the lungs, brain, and liver [11]. The high level of CD44 expression by CSCs has been highlighted as one possible contributor, as both hyaluronan and osteopontin (OPN), common ligands for CD44, are expressed in the bone and other common sites of metastasis [36], suggesting a possible adhesive interaction for circulating tumor cell arrest. *In vitro*, the CD44-hyaluronan interaction has been shown to mediate the attachment of metastatic breast cancer cells to human bone marrow endothelial cells [37]. Moreover, this interaction could be abrogated through the depletion of CD44 expression using RNA interference and induced by the transfection of a CD44^low^ breast cancer cell line with CD44 expression vectors [37]. Additionally, breast cancer cell lines exhibit different levels of Chemokine (C-X-C motif) Receptor 4 (CXCR4), which appears to positively correlate with both CSC proportions and the propensity of breast cancer cell lines to metastasize [18, 38]. Similar observations were made in pancreatic cancer, where within the identified CD133^+^ CSC population, there existed two subpopulations based on CXCR4 expression, and only the CXCR4^+^ population was capable of metastasizing [39]. Although the mechanisms have not yet been elucidated, there is evidence to suggest that CSCs are not only tumor-initiating cells, but also metastasis-initiating cells (M-ICs). The role of CSCs in driving organ tropism of breast cancer remains to be determined. 

Recent work has also highlighted that CSCs isolated from tumors originating in the breast and other tissues exhibit resistance to chemotherapy and radiation [40–43]. A study of human leukemia revealed that the chemoresistance of leukemic CSCs arises from the quiescent nature of these cells, as they are stationary in the G_0_ phase, which limits the effectiveness of chemotherapeutics that target actively replicating cells [44]. In humans, an increase in the proportion of CD44^+^ CD24^−^ breast cancer cells has been observed after neoadjuvant chemotherapy, indicating likely CSC therapy resistance *in vivo* [19]. Possible mechanisms for this include the expression of cell surface drug efflux pumps, such as breast cancer resistance protein-1 (BCRP1; ABCG2), which are capable of expelling chemotherapeutic drugs [45]. Interestingly, BCRP1 is also highly expressed in normal hematopoietic stem cells [46]. Additionally, the presence and activity of ALDH, an enzyme that is capable of metabolizing and inactivating cytotoxics such as cyclophosphamide [47], is likely playing a key role in the observed chemoresistance. Other factors potentially prolonging the lifespan of CSCs include the increased expression of antiapoptotic molecules such as Bcl-2 and survivin [48, 49]. It remains unclear whether this observed metastatic ability and resistance to therapy is a property attributable only to the CSCs (i.e., innate therapy resistance), or whether these specialized cells also receive signals from their microenvironment in the secondary organ that enhance their survival and resilience in the face of cytotoxic treatment. New therapeutic targets may therefore emerge as we gain a greater understanding of the organ-specific interactions between tumor cells (the “seeds”) and secondary organ sites (the “soil”). 

## 6. CSCs and the Metastatic Microenvironment

There are two prevailing schools of thought as to the origin of the CSC: either (1) a CSC may originate from a normal tissue stem cell (SC) that has acquired tumorigenic mutations; or (2) a CSC may originate from a more differentiated progenitor/mature cell that has dedifferentiated and adapted a stem-like phenotype. Both theories remain under investigation. Recent work by Gupta et al. supports the latter theory by demonstrating that subpopulations within the SUM149 and SUM159 breast cancer cell lines are capable of interconversion between stem-like, basal, and luminal populations. They demonstrate that a phenotypic equilibrium is consistently reached over time both *in vitro *and *in vivo*, although the *in vivo* growth requires coinjection of basal or luminal cells with irradiated carrier cells to allow for these two subtypes to persist long enough to give rise to stem-like cells [50]. The rate at which this interconversion occurs depends only on the current subpopulation of a cell and is not influenced by the history of the cell. In support of this, Scaffidi and Misteli successfully generated CSC-like and non-CSC-like cells after oncogenic reprogramming of differentiated fibroblasts. They observed a stochastic emergence of a small population of CSC-like cells expressing stage-specific embryonic antigen 1 (SSEA-1), a marker that did not arise in any of their control lines, suggesting that the CSC phenotype may occur spontaneously after the main oncogenic events have occurred [51]. Further work that supports this “dedifferentiation” of non-CSCs into CSCs demonstrates the possibility that IL-6 may be a key mediator of the process [52] and highlights the need for further investigation into the origin of CSCs and the effects of their microenvironment on regulating this cellular plasticity.

Regardless of their origin, the functional similarities between CSCs and normal SCs are striking. Normally, the SC niche provides signals that either maintain SC quiescence, promote symmetrical division leading to self-renewal, or promote asymmetrical division leading to differentiation and progression down the lineage [53]. Interactions between SCs and their niche are highly dynamic and essential for proper function [54]. As SCs depend on the surrounding microenvironment for important signals, it is not unreasonable to hypothesize that CSCs may also rely on their microenvironment to maintain their tumor-initiating and metastasis-initiating capacity and that a “metastatic niche” may exist in those organs in which these cells are more likely to create metastatic lesions. This niche may play an important role in the organ tropism observed in breast and other cancers. Additionally, signals from the metastatic niche may cause the interconversion of non-CSCs that have arrived from the primary tumor into more metastatic CSCs.

## 7. Seed and Soil Interactions in the Metastatic Niche

**Figure 1:**
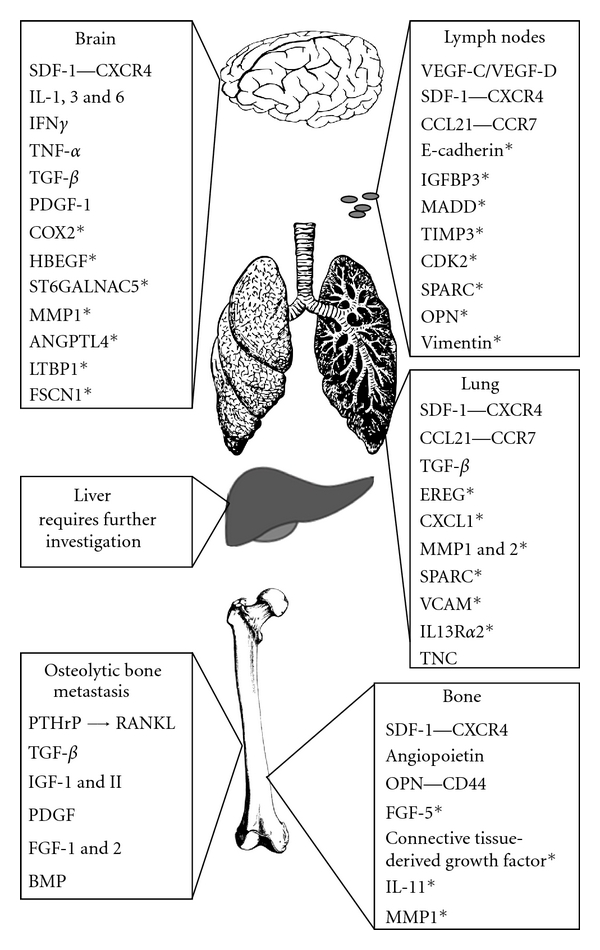
Potential factors involved in the organ-specific metastasis of breast cancer to the brain, liver, lymph nodes, lung, and bone. Brain, lung, and liver images were acquired with thanks to Creative Commons Licensing (CC0 1.0, Public Domain Dedication). Bone image from Gray's Anatomy (1918, Public Domain, copyright expired). Underlining indicates tumor-derived factors. *Italics* indicate organ-derived factors. *indicates factor identified by microarray analysis of organ-specific metastatic cell line variants [56–59].

In the bone marrow, there are functionally different hematopoietic stem cell niches depending on physical location [53, 55]. Synonymously, the metastatic niches around the body may vary, thus dictating what types of cancer cells will be successful in various secondary organs and contributing to the observed organ tropism of different cancer types. The next part of this paper summarizes what is currently known about the metastatic microenvironments provided by common sites of breast cancer metastasis, including bone, brain, lung, liver, and lymph node (Figure 1). 

### 7.1. Bone

Bone is one of the main sites of metastasis for breast cancer, and many groups postulate that this is due to the rich nature of the niche, as it is already optimized for support of normal hematopoiesis [60, 61]. Bone cells express high amounts of stromal-derived factor 1 (SDF-1), which may allow for breast cancer cell migration in a CXCR4^+^-dependent manner [62]. Additionally, the bone microenvironment is rich in ligands such as OPN, which may further support CSC recruitment to the bone through interactions between tumor cell-surface receptors such as CD44 [36, 55]. When a breast cancer cell line variant was selected *in vivo* for increased metastatic capacity for bone, genotypic analysis revealed the upregulation of many genes relative to those expressed by an adrenal medulla seeking variant of the same cell line, including CXCR4, fibroblast growth factor-5 (FGF-5), connective tissue-derived growth factor, interleukin-11 (IL-11), and matrix metalloproteinase 1 (MMP1). This suggests that these cells have innate capabilities to interact with the bone microenvironment, including promotion of both angiogenesis and osteolysis through the differentiation of osteoclasts or cleavage of collagen [57].

Once in the bone, tumor cells exert a profound effect on the bone microenvironment, known as the “vicious cycle” [61]. Normally, the bone is a highly dynamic structure, constantly undergoing remodeling in a carefully regulated balance of osteoblast-mediated bone formation and osteoclast-mediated bone resorption. Breast cancer commonly causes osteolytic bone metastases, indicating the balance has shifted in favor of bone degradation. In a clinical study of breast cancer metastases to the bone, 92% of bone metastases scored high by immunohistochemistry for parathyroid-hormone-related protein (PTHrP) compared to 17% in nonbone sites [63], an observation that was further supported by similar *in situ* hybridization results [64]. It is thought that PTHrP plays an important role in mediating osteolytic bone metastases [65]. The secretion of PTHrP causes osteoblasts to increase their expression of the membrane protein receptor activator of nuclear factor *κ*B (RANK) ligand (RANKL), which promotes osteoclast precursor differentiation and activation through RANK activation [66]. Degradation of the bone matrix causes the release of growth factors, including transforming growth factor-*β* (TGF-*β*), insulin-like growth factors I and II (IGF-I and II), platelet-derived growth factor (PDGF), FGF-1 and -2, and bone morphogenic proteins (BMP), all of which have effects on both osteoblasts and tumor cells [67], causing an increase in tumor cell secretion of PTHrP and the propagation of the vicious cycle. Additionally, these growth factors enter the systemic circulation where they have potential to stimulate cells at distant sites, potentially creating additional metastatic niches and permitting tumor spread. Interestingly, in a large prospective study involving 526 patients afflicted with operable breast cancer, Henderson et al. found that positive PTHrP staining in the primary tumor correlated with an improved survival in 79% of cases, contrary to expected results [68]. These results highlight the need for further investigation of the interaction between breast cancer cells and the bone microenvironment as it appears to be more complex than originally thought.

### 7.2. Brain

The brain represents a unique metastatic niche. It is judiciously guarded by the blood-brain barrier (BBB), a continuous sheet of nonfenestrated endothelium joined by tight junctions and supported by a basement membrane, pericytes and astrocytes [69]. These endothelial cells are armed with ATP-binding cassette C1 (ABCC1) and P-glycoprotein (PGP/ABCB1) and are thus capable of active efflux of most chemotherapeutic drugs from the brain parenchyma [70]. The mechanism by which tumor cells traverse the BBB is poorly understood, but it is postulated that tumor cells adhere to the endothelium and promote endothelial retraction to expose the basement membrane and allow for tumor cell invasion [71]. 

Brain metastases are associated with later stages of disease progression and often only occur secondary to other metastatic lesions in the bone, lung, and/or liver [72]. Thus, brain metastases may potentially represent the manifestation of the true metastatic cascade, or metastasis of metastases [10]. This theory suggests that primary tumor cells first colonize a visceral organ or regional lymph node before acquiring the phenotype necessary to successfully traverse the BBB and interact with the brain microenvironment. Once inside the brain parenchyma, tumor cells encounter a rich microenvironment of cytokines and growth factors, predominantly produced by astrocytes (i.e., SDF-1*α* [73], IL-1, IL-3, IL-6, interferon-*γ* (IFN-*γ*), tumor necrosis factor-*α* (TNF-*α*), TGF-*β*, and PDGF-1 [74]), which the tumor cells usurp to promote survival, growth, and potentially organ-specific metastasis [62]. Furthermore, astrocytes have been shown to exert a tumor-protective effect from chemotherapeutics via direct cell-cell contact [75]. It is likely that a combination of these factors contributes to the highly resistant nature of brain metastases to therapeutics and must be taken into account for the development of new therapeutics.

Further insight into the interactions between tumor cells and the brain microenvironment has been elegantly demonstrated by isolation of a brain-specific metastatic variant of the MDA-MB-231 human breast cancer cell line through repeated selection *in vivo *by Bos and colleagues [56]. Genetic comparison with the parental line highlighted increased expression in the brain variant of cyclooxygenase-2 (COX2), heparin-binding EGF (HBEGF), and sialyltransferase ST6 (alpha-N-acetyl-neuraminyl-2,3-beta-galactosyl-1,3)-N-acetylgalactosaminide alpha-2,6-sialyltransferase 5 (ST6GALNAC5) as potential facilitators of tumor-cell passage through the BBB. Additionally, the authors highlighted collagenase-1 (MMP1), angiopoietin-like 4 (ANGPTL4), latent TGF-*β*-binding protein (LTBP1), and fascin-1 (FSCN1) as genes that were upregulated in the brain-seeking population and thus, potential mediators of brain metastasis, providing more insight into possible tumor-specific therapeutic targets.

### 7.3. Lung

The physical characteristics of the lung make it an ideal site for colonization and eventual outgrowth of tumor cells. The combination of immense surface area and numerous capillaries make it likely that tumor cells will lodge in the vasculature by sheer mechanical forces. The CXCR4/SDF-1 and chemokine (C-C motif) receptor 7/chemokine (C-C motif) ligand 21 (CCR7/CCL21) interactions may play key roles in accentuating the adhesion of tumor cells as the lung endothelium expresses a high level of SDF-1 and CCL21 to complement tumor cell expression of CXCR4 and CCR7 [62, 76]. Additionally, the growth factor transferrin has been suggested to have protumor effects on cells that have the potential to metastasize to the lung but not to their nonmetastatic counterparts [77]. In a Neu-induced transgenic mouse model of breast cancer, TGF-*β* functioned to promote lung metastases [78], in agreement with the well-established multifunctionality of TGF-*β* as being a tumor suppressor in the early stages of cancer, but a metastatic promoter in late stages [79].

Genetic analysis of a lung-specific metastatic variant of the MDA-MB-231 human breast tumor cell line has identified several genes that appear to mediate successful lung metastasis. Minn et al. [58] highlight a combination of secretory and receptor proteins including EGF family member, epiregulin (EREG), CXCL1, MMP1 and 2, cell adhesion molecules secreted protein acidic and rich in cysteine (SPARC; osteonectin) and vascular cell adhesion molecule 1 (VCAM1), and the IL-13 decoy receptor IL13R*α*2. Further analysis of this lung-targeting variant has highlighted the increased expression of tenascin C (TNC) when compared to the parental MDA-MB-231 line. TNC is a component of the extracellular matrix, and the authors suggest that tumor-secreted TNC plays an important role in determining the metastasis initiating capacity of a cell [59]. While there is some overlap between gene expression profiles of organ-specific variants of the same cell line, enough of a discrepancy exists that there are clear lung, bone, and brain metastasis signatures.

### 7.4. Liver

The prevalence of liver metastases in colon cancer far exceeds that of breast cancer, which has resulted in more research being done on the former. Consequently, identified interactions between colon cancers and hepatic metastases may not apply to breast cancers. However, hints about the metastatic mechanisms of breast cancer do arise in the observation of liver colonization by breast cancer cells. In a study by Stessels et al., 43 out of 45 breast cancer cases examined with liver metastases exhibited what is known as replacement growth, where tumor cells displace hepatocytes to coopt the sinusoidal blood vessels while preserving liver architecture [80]. This method of colonization allows for tumor growth independent of angiogenesis. To date, liver-targeting breast cancer cell line variants have not been established, but once selected for, genetic comparison between the organ-specific variants mentioned above will provide invaluable insight into the mechanisms driving liver-specific metastatic disease.

### 7.5. Lymph Nodes

In addition to hematogenous dissemination, breast cancer cells may also metastasize via the lymphatic system. Metastatic tumor cells may either stimulate lymphangiogenesis and enter the nascent vessels or may invade into preexisting lymphatic vasculature. Important primary tumor-derived signals may stem from the VEGF-C/VEGF-D activation of lymphatic endothelial VEGFR-3, which stimulates lymphangiogenesis toward the primary tumor and allows for cellular dissemination [81]. Conversely, molecules proposed to be direct mediators of lymphatic colonization include CCL21 and SDF-1 interacting with their tumor-expressed receptors, CCR7 and CXCR4, respectively. These pairs play important roles in the physiologic homing of lymphoid or hematopoietic cells, and their ligands are highly expressed in the lymph nodes. Additionally, blocking of the CXCR4-SDF-1 interaction with a neutralizing antibody in an *in vivo* model of breast cancer metastasis successfully blocked metastases to the axillary lymph nodes [62].

A lymph node specific variant (468LN) of the MDA-MB-468 breast cancer cell line has been isolated and its mRNA expression compared to a variant of low lymphatic metastatic capacity (468GFP) [82]. When genes identified by differential expression were further compared to gene sets identified through clinical observations to ensure relevance, pathways associated with cell survival and growth in foreign environments were highlighted. Of note, E-cadherin, insulin-like growth factor binding protein 3 (IGFBP3), MAP-kinase activating death domain (MADD), and tissue inhibitor of metalloproteinase 3 (TIMP3) were downregulated, while cyclin-dependent kinase 2 (CDK2), SPARC, OPN, and vimentin were all upregulated. Additionally, the 468LN line harbored a larger CD44^+^ CD24^−^ population (96.4%) than the 468GFP line (6.3%) suggesting a role for breast CSCs in mediating this metastatic capacity.

The factors that have been discussed above for the various metastatic niches represent a brief summary of what is known and are not exhaustive. The diversity of the potential interactions between seed and soil highlights the need for further research. In particular, the question of whether the presence of the primary tumor can influence microenvironmental changes in distant organs prior to tumor cell arrival and metastatic colonization is intriguing.

## 8. Prepping the “Soil”: The Premetastatic Niche

Recent work has shown that primary tumors may play an important role in creating a “premetastatic niche” prior to cancer cell arrival at secondary sites. Work by Kaplan et al. [83] highlighted the role of vascular endothelial growth factor receptor-1 positive (VEGFR1^+^) hematopoietic progenitor cells (HPCs) in the creation of this niche. When signals from the primary tumor tip the normal balance between pro- and antiangiogenic signals in favor of angiogenesis, the angiogenic switch is triggered, causing the recruitment of new vessels to the tumor site [84]. During this process, HPCs are mobilized and migrate towards the tumor-specific premetastatic niche where they form clusters. Characterization of these cells revealed conserved progenitor markers of CD133, CD34, CD117 (c-Kit) in addition to expression of very late antigen-4 (VLA-4; integrin *α*4*β*1), suggesting a VLA-4-fibronectin interaction between migrating HPCs and the new microenvironment. Additionally, MMP9 was expressed by the premetastatic clusters, potentially due to integrin-dependent activation of VEGFR1^+^ HPCs, thereby altering the microenvironment through the breakdown of basement membranes and resultant release of soluble Kit-ligand. This study further showed that the VEGFR1^+^ cells supported tumor cell adherence and growth and that metastasis could be abrogated upon the treatment with an anti-VEGFR1 antibody, highlighting the importance of these clusters in the creation of the premetastatic niche [83]. 

Another method that tumors use to condition the metastatic niche relies on microvesicular (MV) deposition of factors. Tumor-derived MVs, or exosomes, are derived from the inner membranes of the late endosomes and range from 40 to 100 nm in diameter. Release into the surrounding tissue or bloodstream occurs when the endosomes fuse with the cellular membrane [85]. Although the underlying mechanism is not fully understood, MVs may stimulate target cellular receptors directly, transfer surface receptors from cell to cell, deliver proteins [86], or may even cause epigenetic reprogramming of cells [87]. Additionally, MVs have been found to harbor immunosuppressive molecules [88]. Thus, exosomes may provide important signals to the tumor cells once they arrive in the metastatic niche, in addition to sculpting the stromal and immune cells systemically. 

A recent concern arising from the revelation that exosomes are functional moieties and not just carriers of cellular waste arises from the potential for horizontal gene transfer between tumor cells and bone-marrow-derived cells (BMDCs) recruited to the premetastatic niche. Lyden and colleagues call this phenomenon “tumor exosome-driven education” of BMDCs [89]. This process likely promotes the progrowth and survival environment of the niche and may potentiate the metastatic process. Given their multifunctionality, it is likely that tumor-derived exosomes contribute to the creation of the premetastatic niche. Therefore, although the immunosuppressive effects of exosomes must first be negated, exosomes may represent a novel cell-free source of tumor antigens that can be utilized in the creation of an anti-cancer immunization to enhance the anti-tumor immune response [90]. 

## 9. The Cancer Stem Cell Niche: Does It Exist?

To date, published literature has used whole cell populations of organ-specific metastatic variants of human tumor cell lines as a model to investigate the organ tropism of metastasis [56–58]. However, these studies have overlooked the involvement of CSCs in this process. Further characterization of the distinct subpopulation of CSCs within these metastatic variants is needed to see if more refined genetic signatures can be obtained, possibly dictating a more specific niche for metastasis. If CSCs are indeed the initiators of metastasis, it is important to determine if these cells also exhibit organ-specific behaviors or if they are innately more metastatic to all sites in a nonspecific manner. Further investigations could also include murine models of spontaneous metastasis utilizing CSC and non-CSC subpopulations to elucidate if both subpopulations equally recruit the VEGFR1^+^ population observed by Kaplan et al. [83] to the premetastatic niche, or if this capacity resides within one subpopulation. Our lab has observed increased tumorigenicity and metastatic ability to the lung of stem-like ALDH^hi^ CD44^+^ stem-like breast cancer cells relative to nonstem-like ALDH^low^ CD44^−^ cells [18]. This observed metastatic proficiency of CSCs may be partially attributed to their ability to create the premetastatic niche, in addition to their ability to form significant primary tumors. However, the exact mechanism behind this increased metastatic potential remains unknown. Additional characterization of the cell surface molecules expressed by CSCs may also provide further insight into their roles in metastatic organ tropism. For example, CSC expression of receptors such as CXCR4 would confer specific targeting to areas where SDF-1 is highly expressed, such as bone, lung, lymph node, and brain [62, 73, 76], where the cells would then receive additional signals to support colonization. Additionally, CSCs may express higher levels of cell-surface receptors than their non-CSC counterparts so that they may fully harness the soluble growth factors present at secondary sites, conferring a growth advantage and permitting successful colonization.

## 10. Therapeutic Implications/Conclusions

A better understanding of the mechanism underlying the metastatic process is needed in order to increase the efficacy of treatments against this lethal process of disease progression. Metastatic lesions are often highly resistant to therapies, possibly due to the resident CSCs. In breast cancer, it would appear that the purported CSC subpopulation also encompasses the metastasis-initiating population. A better understanding of the interactions between CSCs and host organs may therefore lead to the identification of new targets that may allow for the abrogation of metastatic growth signals and consequently successful targeting of metastatic disease. Conversely, innate inhibitory factors may be found in the hostile secondary organs that may also be harnessed for therapeutic purposes. The definition of the microenvironment has evolved to include soluble factors, extracellular matrix, cell surface molecules, chemokines, hormones, and now exosomes, widening the scope of interactions that must be investigated. 

There is no question that the clinically observed patterns of metastasis are relevant for cancer therapy, as there must be specific organ-cancer cell interactions contributing to the viewed success and failures of cancer cells to colonize specific secondary sites. In addition to targeting tumor-secreted factors, research is needed to identify key innate factors providing attractive and/or growth signals for the arriving cancer cells, so that inhibitors or specific targeting molecules may be developed against these factors. Furthermore, elucidation of the role of CSCs in this metastatic organ-tropism is also important, as new therapies are required to target this innately therapeutic resistant subpopulation. In light of the potential for interconversion between non-CSCs and CSCs, new therapies must target both populations of cells to be effective.

Further understanding of the role of CSCs in metastasis can be acquired with the characterization of circulating tumor cells (CTCs). Research in the CTC field is rapidly developing, and innovative techniques for the capture and characterization of CTCs are rapidly evolving. The many platforms to date (eloquently reviewed by Lowes et al. [91] and Yu et al. [92]) allow researchers to choose their method of capture based on either molecular cellular characteristics such as epithelial cell adhesion molecule (EpCAM)^+^CD45^−^Cytokeratin 8, 18, and 19^+^ (CellSearch; Veridex), EpCAM^+^ (microfluidic CTC-chip [93, 94]), or markers of the researcher's choice (Fiber-optic array scanning technology [95, 96]), or physical cell size (filter-based platforms [97, 98]). Regardless of the platform, these techniques will allow for the further characterization of CTCs providing insight into the mechanisms driving organ tropism and whether CSCs are involved. Additionally, CTC data will offer distinct benefits for individualized therapy, as physicians could tailor therapy to the characteristics of the CTCs.

As the world's population ages, the incidence of cancer is projected to increase, making more effective treatments vital to help combat this growing world-wide burden. Although methods for early detection are in place for more developed countries, these capacities are not readily available in developing countries. Thus, cancers in these areas will often be detected during the later stages of disease progression, when metastasis has likely already occurred. Novel, more effective metastatic treatments may be the only option for this new group of cancer patients and are already desperately required for those in developed countries burdened with metastatic breast cancer. In addition to further understanding the characteristics of cancer stem cells, future research should focus on the interactions between CSCs and the secondary organs of metastasis, as we believe this to be where new metastatic targets will arise.

## Figures and Tables

**Table 1:** Interactions between primary cancer site and target organ based on metastatic efficiency indexes.

Primary cancer site	Target organ							
	Kidney	Brain	Bone	Skeletal muscle	Skin	Heart	Thyroid	Adrenal
Bone	—	—	—	—	—	/	—	↑
Breast	—	—	↑	—	—	—	↑	↑
Cervix	—	—	—	—	↓	/	↑	↑
Colorectal	—	—	—	↓	—	—	—	↑
Esophagus	—	—	—	—	↓	/	↑	↑
Kidney	—	—	—	↓	—	—	↑	↑
Lung	—	—	—	/	/	—	—	↑
Lung(SCC)	—	—	—	/	/	/	—	↑
Osteosarcoma	↓	↓	—	↓	/	↓	—	↓
Ovary	↓	↓	—	/	—	—	↑	↑
Ovary*	—	↓	—	—	—	/	—	↑
Pancreas	—	—	—	—	—	—	—	↑
Prostate	—	—	↑	—	↓	↓	—	↑
Prostate*	—	↓	↑	/	↓	—	—	↑
Stomach	—	↓	—	↓	—	/	—	↑
Testis	—	—	—	↓	—	/	—	↑
Thyroid	—	—	—	—	—	/	—	↑
Urinary Bladder	—	↓	—	—	—	/	↑	↑
Uterus	—	—	—	—	↓	/	↑	↑

Adapted from Weiss (1992) [10].

↑ Friendly (Increased incidence) (MEI > 0.100).

↓ Hostile (Decreased incidence) (MEI < 0.009).

— Neutral (0.010 < MEI < 0.099).

/ Not reported.

SCC: small cell carcinoma.

*Duplicate sites due to different autopsy studies used.

## Abbreviations

ABCB1: ATP binding cassette subfamily B member 1
ABCC1: ATP binding cassette subfamily C member 1
ABCG2: ATP binding cassette subfamily G member 2
ALDH: Aldehyde dehydrogenase
ANGPTL4: Angiopoietin-like 4
BBB: Blood-brain barrier
BCRP1: Breast cancer resistance protein-1
BMDC: Bone-marrow-derived cells
BMP: Bone morphogenic protein
CCL21: Chemokine (C-C motif) ligand 21
CCR7: Chemokine (C-C motif) receptor type 7
CD: Cluster of differentiation
CDK2: Cyclin-dependent kinase 2
COX2: Cyclooxygenase-2
CSC: Cancer stem cell
CXCR4: Chemokine (C-X-C motif) receptor 4
EREG: Epiregulin
EpCAM: Epithelial cell adhesion molecule
ESA: Epithelial-specific antigen
FGF: Fibroblast growth factor
FSCN1: Fascin-1
HBEGF: Heparin-binding EGF
HPC: Hematopoietic progenitor cell
IFN-*γ*: Interferon-*γ*
IGF: Insulin-like growth factor
IGFBP3: Insulin-like growth factor binding protein 3
IL: Interleukin
Lin: Lineage
LTBP1: Latent TGF-*β*-binding protein
M-IC: Metastasis-initiating cell
MADD: MAP-kinase activating death domain
MEI: Metastatic efficiency index
MMP: Matrix metalloproteinase
MV: Microvesicle
NOD/SCID: Nonobese diabetic severe combined immune deficiency
OPN: Osteopontin
PDGF: Platelet-derived growth factor
PGP: P-glycoprotein
PTHrP: Parathyroid hormone-related protein
RANK: Receptor activator of nuclear factor *κ*B
RANKL: Receptor activator of nuclear factor *κ*B ligand
SC: Stem cell
SDF-1: Stromal-derived factor-1
SPARC: Secreted protein acidic and rich in cysteine
SSEA-1: Stage-specific embryonic antigen 1
ST6GALNAC5: ST6 (alpha-N-acetyl-neuraminyl-2,3-beta-galactosyl-1,3)-N-acetylgalactosaminide alpha-2,6-sialyltransferase 5
TIMP3: Tissue inhibitor of metalloproteinase 3
TGF-*β*: Transforming growth factor-*β*
TNC: Tenascin C
TNF-*α*: Tumor necrosis factor-*α*
VCAM: Vascular cell adhesion molecule
VEGF: Vascular endothelial growth factor
VEGFR: Vascular endothelial growth factor receptor
VLA-4: Very late antigen-4.
